# Identifying Structural Features of Nucleotide Analogues to Overcome SARS-CoV-2 Exonuclease Activity

**DOI:** 10.3390/v14071413

**Published:** 2022-06-28

**Authors:** Xuanting Wang, Chuanjuan Tao, Irina Morozova, Sergey Kalachikov, Xiaoxu Li, Shiv Kumar, James J. Russo, Jingyue Ju

**Affiliations:** 1 Center for Genome Technology and Biomolecular Engineering, Columbia University, New York, NY 10027, USA; xw2467@columbia.edu (X.W.); ct2439@columbia.edu (C.T.); im198@columbia.edu (I.M.); sk363@columbia.edu (S.K.); lx2109@columbia.edu (X.L.); sk3765@columbia.edu (S.K.); jjr4@columbia.edu (J.J.R.); 2 Department of Chemical Engineering, Columbia University, New York, NY 10027, USA; 3 Department of Molecular Pharmacology and Therapeutics, Columbia University, New York, NY 10032, USA

**Keywords:** SARS-CoV-2, coronaviruses, antivirals, RNA-dependent RNA polymerase, exonuclease, nucleotide analogues

## Abstract

With the recent global spread of new SARS-CoV-2 variants, there remains an urgent need to develop effective and variant-resistant oral drugs. Recently, we reported in vitro results validating the use of combination drugs targeting both the SARS-CoV-2 RNA-dependent RNA polymerase (RdRp) and proofreading exonuclease (ExoN) as potential COVID-19 therapeutics. For the nucleotide analogues to be efficient SARS-CoV-2 inhibitors, two properties are required: efficient incorporation by RdRp and substantial resistance to excision by ExoN. Here, we have selected and evaluated nucleotide analogues with a variety of structural features for resistance to ExoN removal when they are attached at the 3′ RNA terminus. We found that dideoxynucleotides and other nucleotides lacking both 2′- and 3′-OH groups were most resistant to ExoN excision, whereas those possessing both 2′- and 3′-OH groups were efficiently removed. We also found that the 3′-OH group in the nucleotide analogues was more critical than the 2′-OH for excision by ExoN. Since the functionally important sequences in Nsp14/10 are highly conserved among all SARS-CoV-2 variants, these identified structural features of nucleotide analogues offer invaluable insights for designing effective RdRp inhibitors that can be simultaneously efficiently incorporated by the RdRp and substantially resist ExoN excision. Such newly developed RdRp terminators would be good candidates to evaluate their ability to inhibit SARS-CoV-2 in cell culture and animal models, perhaps combined with additional exonuclease inhibitors to increase their overall effectiveness.

## 1. Introduction

SARS-CoV-2, the causative agent of COVID-19, is a member of the Nidovirales order of positive-strand RNA viruses [1]. Members of this coronavirus family include those responsible for SARS, MERS and assorted mild respiratory infections in humans and animals [2]. The coronaviruses fall into four major groups, designated alpha, beta, gamma and delta [3]; SARS-CoV, MERS-CoV and SARS-CoV-2 are in the beta lineage and are closely related to one another [4]. Like other coronaviruses, SARS-CoV-2 has a large RNA genome encoding more than 25 proteins. There are 16 nonstructural proteins (Nsp1-16), many of which form the replication-transcription complex (RTC). The functions of these proteins have been extensively reviewed [5,6], and several of them have been selected as targets for drug development.

Because of the large genome size of the coronaviruses (>30 kb), their relatively low fidelity RNA-dependent RNA polymerase (RdRp) would tend to produce a high number of errors during RNA replication and transcription [7]; the resulting mutations could impede viral replication and infectivity. To overcome this, the coronaviruses possess a 3**′**-5**′** exonuclease (ExoN), consisting of Nsp14 and Nsp10, involved in proofreading and error repair [7,8,9,10,11,12,13,14,15,16,17], along with other functions such as evasion of host immunity [18]. The RdRp and ExoN of SARS-CoV-2 work together in lockstep to ensure that mismatches and modified nucleotides mistakenly incorporated by the RdRp are efficiently removed by ExoN, and ExoN knockout mutants of SARS-CoV-2 are nonviable [14]. 

The complete structure of the RTC (Nsp12/7/8, Nsp14/10, Nsp13 helicase, and associated proteins Nsp9 and Nsp16), as well as that of the individual components of the complex, has been resolved by cryo-electron microscopy and X-ray crystallography in order to determine which sites in these proteins are critical for protein-protein interactions or catalytic activity. The structures determined include the replicating RdRp complex in the absence and presence of RNA and specific nucleotide inhibitors [19,20,21,22,23,24]; the ExoN complex, including in the presence of mismatched bases, implicating a variety of proofreading mechanisms including backtracking [11,25]; and the complete and near-complete RTC that includes helicase and capping enzymes [26,27,28,29,30].

The Nsp14 protein contains an ExoN functional domain that provides replication and transcription error correction, allowing SARS-CoV-2 to maintain its large-sized genome [12,31,32,33,34]. Enzymatic assays confirm that the accessory protein Nsp10 stabilizes substrate binding by ExoN to support its exoribonuclease activity [13]. The Nsp14 and Nsp10 residues involved in these interactions have been studied using 3D structure analysis and artificial mutagenesis. Artificial mutations introduced into Nsp14 helped identify the positions most important for its function [12,35,36]. Specific residues were identified in SARS-CoV that might be targeted for preventing Nsp14-Nsp10 interactions and activity, with possible negative consequences for viral replication [37,38]. The activity of Nsp14 has recently been reported to be enhanced by Nsp8 [7].

Recently, various assays have been described to measure the SARS-CoV-2 ExoN activity [7,15,39,40]. Based on molecular assays with reconstituted enzyme complexes (Nsp14/Nsp10), the ExoN enzyme can recognize and remove mismatches at the 3′ end as well as internally, the latter suggesting an endonuclease-based repair mechanism distinct from that of the Nsp15 endonuclease [7]. Moreover, Nsp14 can excise not only mismatched natural bases but a wide variety of nucleotide analogues that are incorporated into the RNA [7]. Thus, we and others have reasoned that the well-choreographed dance between the RdRp and the ExoN represents an Achille’s heel for the virus: if we can take advantage of the low fidelity of the RdRp to incorporate modified nucleotides that terminate the RNA polymerase reaction, and at the same time, prevent the removal of these nucleotide analogues by inhibiting the ExoN, we might effectively block SARS-CoV-2 replication [7,39,41]. 

Drugs that are based on nucleoside and nucleotide analogues that inhibit the coronavirus RdRp (Nsp12, Nsp7 and Nsp8) and ExoN (Nsp14 and Nsp10) fall into multiple categories with respect to their modes of action. Several active forms of nucleoside/nucleotide analogues can be incorporated into the viral RNA by the RdRp where they terminate further incorporation or they lead to mutations in the RNA, either of which may impede various aspects of the viral life cycle [39,42,43,44,45,46,47]. However, if they are efficiently excised by ExoN, their effects may be abrogated. For the drug to be effective, its rate of incorporation must significantly exceed its rate of excision, the incorporated nucleotide must be resistant to ExoN, or the ExoN must itself be inhibited by an exonuclease inhibitor [16,41,47,48]. Alternative non-nucleotide-based inhibitors of the RdRp have also been explored as a strategy for interfering with SARS-CoV-2 replication [49,50]. Since these non-nucleotide inhibitors are not incorporated into RNA, they would not be affected by the ExoN excision mechanism.

Previously, we demonstrated that the triphosphate forms of numerous nucleotide analogues, including FDA-approved drugs for other viral infections, are capable of being incorporated into RNA by the SARS-CoV-2 RdRp complex and of terminating RNA extension with varying efficiency, in either immediate or delayed fashion [46,47]. Minskaia et al. [51] reported that the SARS-CoV ExoN did not hydrolyze DNA or ribose-2′-O-methylated RNA substrates. We identified several hepatitis C virus (HCV) NS5A inhibitors [52] that also inhibited the SARS-CoV-2 exonuclease [41,53,54]. Several of these ExoN inhibitors acted synergistically with RdRp inhibitors in blocking viral replication in Calu-3 cells [41]; recently, other investigators have proposed similar combination drug approaches [55,56]. Interestingly, our studies indicated that the HCV NS5A inhibitors Velpatasvir and Daclatasvir not only inhibited the exonuclease but inhibited RdRp activity as well [53,54]. We also noticed that Tenofovir, once incorporated into RNA by RdRp, was largely resistant to removal by ExoN [41]. This led us to examine further the properties of nucleotide analogues that might lead to such ExoN resistance. 

**Figure 1:**
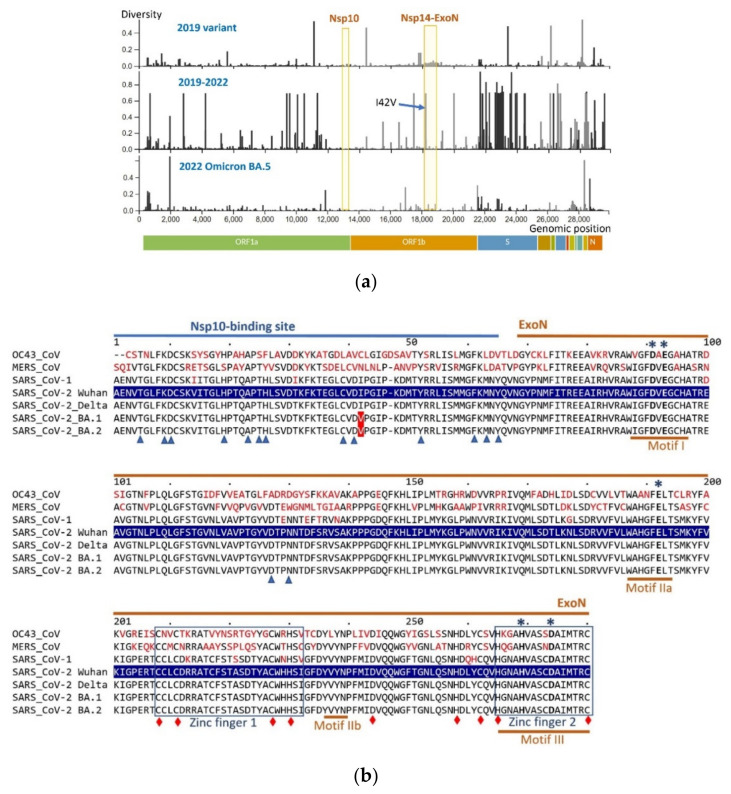
(**a**) **Diversity of the encoded amino acids along genomic sequences of SARS-CoV-2.** Profiles for three genomic sets are shown—**top:** diversity within ~2000 genomic sequences of the early SARS-CoV-2 sampled between December 2019 and March 2020; **middle:** diversity within ~2000 genomes collected in December 2019–May 2022; **bottom:** diversity within ~900 genomes of the recent Omicron BA.5 variant collected from January–May of 2022. The Nsp14-ExoN boundaries include only the first 280 aa positions involved in ExoN function (the same region shown in the sequence alignment). The arrow points to the position of the ubiquitous I42V aa substitution in Nsp14. Profiles are constructed by the Nextstrain webtool using genomic sequences from GISAID (https://www.gisaid.org/; Nextstrain datasets: “early-19A” and “22B Omicron”; webtool accessed on 25 May 2022 at https://nextstrain.org [61]). (**b**) **Protein sequence conservation in the ExoN domain of Nsp14 in representative beta *Coronaviridae* strains.** Partial Nsp14 protein alignment spans the Nsp10-binding site (amino acid positions 1–64) and the adjacent ExoN domain (aa positions 68–280). Nsp14 of the SARS-CoV-2 Wuhan-Hu-1 variant (highlighted in blue) was used as a reference: amino acid differences in other variants compared to the reference sequence are shown in red. Functional motifs comprising the ExoN active site are underlined, and the zinc finger regions are boxed [12,14,36]. ExoN catalytic residues are indicated with asterisks; residues interacting with Nsp10 are marked by blue triangles; and positions critical for zinc fingers, protein solubility or ExoN activity are shown as red diamonds [12,14]. The sequences were retrieved from GenBank: OC43-CoV (common cold; accession number YP_009924328), MERS-CoV (YP_009047225), SARS-CoV-1 (JX163928), and variants of SARS-CoV-2: Wuhan-Hu-1 (YP_009725309, original strain isolated in 2019), Delta (OM990852), and Omicron BA.1 (ON141240) and BA.2 (ON553707). Protein alignment was built using Clustal Omega [62,63], visualized by MView [63,64], and then annotated.

In this paper, based on published sequence information, we first assess the conservation of protein sequences within the exonuclease functional sites of Nsp14 and Nsp10 in SARS-CoV-2 variants (Figure 1 and Figure S1). The high conservation observed for these proteins indicates that inhibitors of ExoN function would be effective against current and future variants of SARS-CoV-2. Second, we present the results of enzymatic assays evaluating the ability of nucleotide analogues with various structural features to be excised from the 3′ end of RNA by ExoN. We focus on nucleotides and nucleotide analogues with sugar modifications, such as lack of the 2′-OH, lack of the 3′-OH, or lack of both 2′- and 3′-OH, as well as base-modified nucleotides. Among the molecules we tested, nucleotide analogues lacking both the 2′- and 3′-OH moieties were most resistant to ExoN activity. Given the sequence conservation of Nsp14/10 among SARS-CoV-2 variants, the structural features of nucleotide analogues identified above can guide the design and synthesis of novel RdRp inhibitors that can both be efficiently incorporated by the RdRp and substantially resist ExoN excision, perhaps combined with additional exonuclease inhibitors to increase their overall effectiveness to inhibit SARS-CoV-2. 

## 2. Materials and Methods

Nucleoside triphosphate analogues were purchased from TriLink BioTechnologies (Cidofovir-DP, 2**′**-NH_2_-dUTP, Cordycepin-TP, ddATP, ddCTP, ddGTP, Biotin-16-dUTP), Invitrogen (Biotin-16-UTP), Santa Cruz Biotechnology (Stavudine-TP), Amersham Life Sciences (Zidovudine-TP) or Alfa Chemistry (Tenofovir-DP). RNA oligonucleotides (template-loop-primers) were purchased from Dharmacon. HIV reverse transcriptase, SuperScript IV reverse transcriptase and T7 RNA polymerase were purchased from Millipore Sigma, Thermo Fisher and New England BioLabs, respectively. The expression and purification of the SARS-CoV-2 exonuclease Nsp14/Nsp10 complex have been described in our previous study [41]. 

### 2.1. Extension Reactions with Reverse Transcriptase to Produce Nucleotide Analogue-Extended RNA

The RNA template-loop-primers (5**′**-UUUUCUACGCGUAGUUUUCUACGCG-3**′** for Biotin-16-dUTP, Stavudine-TP or Zidovudine-TP extension reactions; 5**′**-UUUUCACCGCGUAGUUUUCUACGCG-3**′** for ddGTP extension reactions; 5**′**-UUUUCAUCGCGUAGUUUUCUACGCG-3**′** for Tenofovir-DP or ddATP extension reactions; 5**′**-UUUUCUGCGCGUAGUUUUCUACGCG-3**′** for Cidofovir-DP or ddCTP) were annealed by heating to 75 °C for 3 min and cooling to room temperature in 1 × HIV reverse transcriptase (RT) or 1 × SuperScript IV RT reaction buffer. Then, 10 μL of the appropriate annealed RNA template-loop-primer solution (10 μM) was added to 8 μL of the reverse transcriptase solution consisting of 54.4 U of HIV RT or 200 U of SuperScript IV RT in 1 × appropriate buffer. Finally, 2 μL of a solution containing 10 mM Biotin-16-dUTP, Stavudine-TP, Cidofovir-DP, Tenofovir-DP, Zidovudine-TP, ddATP, ddCTP or ddGTP was added and incubation was carried out for 4 h at 45 °C. The 20 μL extension reactions contained 54.4 U HIV RT or 200 U SuperScript IV RT, 5 μM RNA template-loop-primer, and 1 mM Biotin-16-dUTP, Stavudine-TP, Cidofovir-DP, Tenfovir-DP, Zidovudine-TP, ddATP, ddCTP or ddGTP. The 1 × HIV RT reaction buffer contained 10 mM Tris-HCl pH 8, 10 mM KCl, 2 mM MgCl_2_ and 1 mM β-mercaptoethanol. Desalting of the reaction mixture was performed with an Oligo Clean & Concentrator kit (Zymo Research), resulting in ~10 μL purified aqueous RNA solutions. Then, 1 μL of each solution was subjected to MALDI-TOF MS (Bruker ultrafleXtreme) analysis. The remaining ~9 μL RNA extension products were used to test exonuclease activity.

### 2.2. Extension Reactions with RNA Polymerase to Produce Nucleotide Analogue-Extended RNA

The RNA template-loop-primers (5**′**-UUUUCUACGCGUAGUUUUCUACGCG-3**′** for 2**′**-NH_2_-dUTP and Biotin-16-UTP; 5**′**-UUUUCAUCGCGUAGUUUUCUACGCG-3**′** for Cordycepin-TP) were annealed by heating to 75 °C for 3 min and cooling to room temperature in 1 × RNA Pol buffer. Then, 10 μL of the appropriate annealed RNA template-loop-primer solution (10 μM) was added to 8 μL of an RNA polymerase solution consisting of 400 U of T7 RNA polymerase in 1 × RNA Pol buffer. Finally, 2 μL of a solution containing 10 mM of 2**′**-NH_2_-dUTP, Biotin-16-UTP or Cordycepin-TP was added and incubated for 4 h at 37 °C. The 20 μL extension reactions contained 400 U T7 RNA polymerase, 5 μM RNA template-loop-primer, and 1 mM 2**′**-NH_2_-dUTP, Biotin-16-UTP or Cordycepin-TP. The 1 × RNA polymerase reaction buffer contains 40 mM Tris-HCl, 6 mM MgCl_2_, 1 mM DTT and 2 mM spermidine. Desalting of the reaction mixture was performed with an Oligo Clean & Concentrator kit (Zymo Research), resulting in ~10 μL purified aqueous RNA solutions. Then, 1 μL of each solution was subjected to MALDI-TOF MS (Bruker ultrafleXtreme) analysis. The remaining ~9 μL RNA extension products were used to test exonuclease activity.

### 2.3. Exonuclease Reactions with SARS-CoV-2 Nsp14/Nsp10 Complex

**Figure 2:**
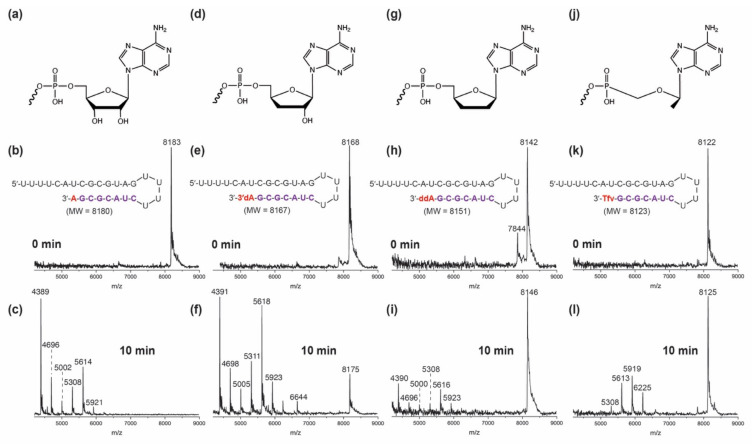
**SARS-CoV-2 exonuclease activity with adenosine- or adenosine analogue-terminated RNA.** A mixture of 500 nM template-loop-primer terminated at its 3′ end with either A (**a**), Cordycepin (**d**), ddA (**g**) or Tenofovir (Tfv) (**j**) (sequences shown in **b**,**e**,**h**,**k**) and SARS-CoV-2 pre-assembled exonuclease complex (Nsp14/Nsp10) was incubated at 37 °C for 10 min. These intact RNAs (**b**,**e**,**h**,**k**) and their respective exonuclease reaction products (**c**,**f**,**i**,**l**) were analyzed by MALDI-TOF MS. The signal intensity was normalized to the highest peak. The accuracy for *m*/*z* determination is approximately ±10 Da.

**Figure 3:**
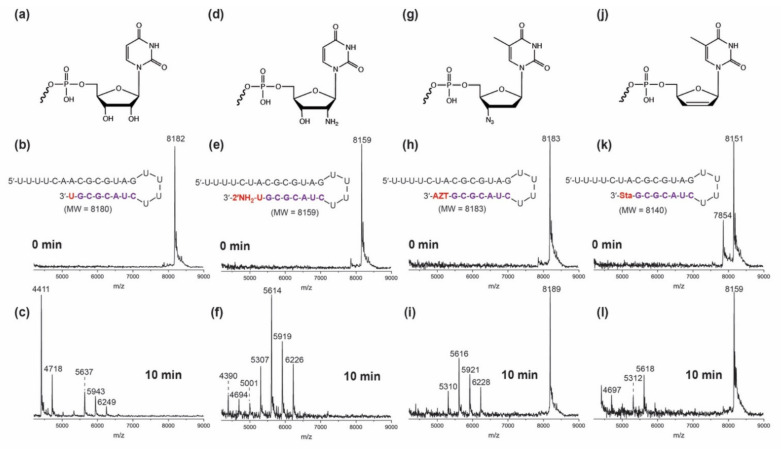
**SARS-CoV-2 exonuclease activity with uridine- or uridine-analogue terminated RNA.** A mixture of 500 nM template-loop-primer terminated at its 3′ end with either U (**a**), 2′-NH_2_-U (**d**), Zidovudine (AZT) (**g**) or Stavudine (Sta) (**j**) (sequences shown in **b**,**e**,**h**,**k**) and SARS-CoV-2 pre-assembled exonuclease complex (Nsp14/Nsp10) was incubated at 37 °C for 10 min. These intact RNAs (**b**,**e**,**h**,**k**) and their respective exonuclease reaction products (**c**,**f**,**i**,**l**) were analyzed by MALDI-TOF MS. The signal intensity was normalized to the highest peak.

**Figure 4:**
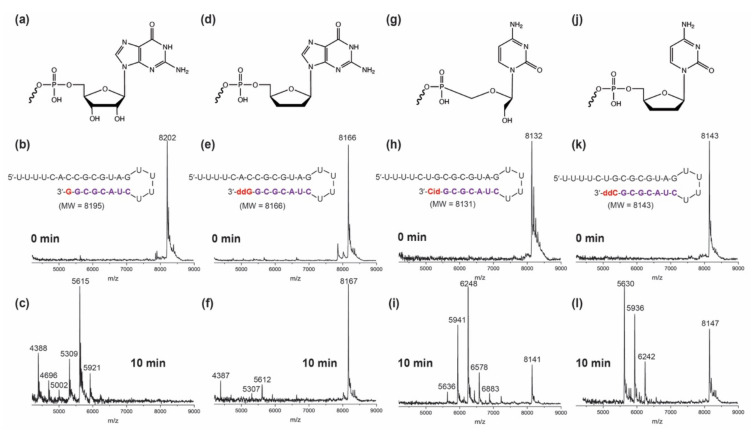
**SARS-CoV-2 exonuclease activity with guanosine- or guanosine analogue-terminated RNA (a–f) and cytosine analogue-terminated RNA (g–l).** A mixture of 500 nM of template-loop-primer terminated at its 3′ end with either G (**a**), ddG (**d**), Cidofovir (Cid) (**g**) or ddC (**j**) (sequences shown in **b**,**e**,**h**,**k**) and SARS-CoV-2 pre-assembled exonuclease complex (Nsp14/Nsp10) was incubated at 37 °C for 10 min. These intact RNAs (**b**,**e**,**h**,**k**) and their respective exonuclease reaction products (**c**,**f**,**i**,**l**) were analyzed by MALDI-TOF MS. The signal intensity was normalized to the highest peak.

The synthetic RNA template-loop-primers with A, U, G, C, dC, 2**′**-F-dC or 2**′**-OMe-C at the 3**′** terminus (sequences shown in Figure 2b,Figure 3b, Figure 4b and Figure S4b,e,h,k respectively) or the extended template-loop-primers with Biotin-16-U, Biotin-16-dU, Stavudine, Cidofovir, Tenofovir, Zidovudine, 2**′**-NH_2_-dU, Cordycepin, ddA, ddC or ddG at the 3**′** end were annealed by heating to 75 °C for 3 min and cooling to room temperature in 1 × exonuclease reaction buffer. To the 10 μL annealed extended RNA template-loop-primer solution (1 μM), 10 μL of exonuclease Nsp14/10 complex (100 nM) in 1 × exonuclease reaction buffer was added and incubated at 37 °C for 5, 10 or 15 min. The final concentrations of reagents in the 20 μL reactions were 50 nM Nsp14/10 and 500 nM different RNAs. The 1 × exonulease reaction buffer contains 40 mM Tris-HCl pH 8, 1.5 mM MgCl_2_, and 5 mM DTT. After incubation, each reaction was quenched by the addition of 2.2 μL of an aqueous solution of EDTA (100 mM). Following desalting using an Oligo Clean & Concentrator (Zymo Research), the samples were subjected to MALDI-TOF MS (Bruker ultrafleXtreme) analysis.

## 3. Results and Discussion

### 3.1. Sequence Conservation in Nsp14 and Nsp10 

With more than 5 million genomes of SARS-CoV-2 sequenced, naturally occurring mutations have been analyzed in a number of strains, including the most recent variants of concern to public health [57,58,59]. Overall, SARS-CoV-2 is a relatively conservative virus compared to other RNA viruses [60]. This low genomic variability means that coronaviral genomes contain lower numbers of mutations, and analysis of those mutations that become widespread may help in our understanding of viral functions. In the coronaviruses, low genome variability is ensured by the activity of ExoN during RNA replication [7,9,11,12,14,15]. 

The majority of genomic sequence variations observed in SARS-CoV-2 variants are located within genes encoding Spike, structural and accessory proteins, while the members of the ExoN complex, Nsp14 and Nsp10 genes, accumulate fewer nucleotide changes, most of which are synonymous (not causing changes in the encoded amino acids). Figure 1a shows amino acid diversity profiles for an early 2019 SARS-CoV-2 strain and the recent Omicron BA.5 variant. Nsp10 displays high sequence conservation. Moving from early variants to more recent ones, the overall amino acid diversity in Nsp14 becomes lower, in high contrast with the increasing sequence diversity in the Spike protein, for example. Within the ExoN-related portion of Nsp14, only one amino acid position retains diversity, displaying a conservative substitution I42V. Such sequence conservation in Nsp14 and Nsp10 may reflect a negative evolutionary pressure on the structure of the protein components of the ExoN complex, preserving its function, along with a bottleneck effect accounting for the widespread distribution of the neutral I42V.

Figure 1b shows the primary structure of the exonuclease-related domains of the Nsp14 protein, in four SARS-CoV-2 strains (the original 2019 isolate Wuhan-Hu-1, Delta, Omicron BA.1 and Omicron BA.2), along with SARS-CoV-1, MERS-CoV and OC43-CoV, a coronavirus causing the common cold. Positions marked with asterisks, triangles and diamonds in Figure 1b correspond to amino acid residues that are critical for ExoN function. Artificially introduced mutations at these positions have been shown to hinder the ExoN proofreading activity [12,14,36]. The catalytic residues at positions marked with asterisks comprise the active center of the ExoN domain of Nsp14. 

When recent variants of SARS-CoV-2 are compared to the original 2019 strain, very few mutations in the replication component, including Nsp14 and Nsp10 proteins, became fixed in the population. Within the portion of Nsp14 that spans the Nsp10-binding site and ExoN domain (Figure 1b), the only widespread naturally occurring mutation is I42V, a conservative substitution in the Nsp10-binding site, as evidenced by analysis of more than 80,000 SARS-CoV-2 genomes [59]. This is the only mutation that is ubiquitous in the exonuclease-related portion of Nsp14 in Omicron sub-lineages [60]. Another amino acid change in Nsp14, a D to N substitution at amino acid position 212 in the Figure 1b alignment (mutation not shown), present in some BA.2 sequences (e.g., GenBank accession ON117774), is located in the first zinc finger region of the ExoN domain. This substitution is between similar polar amino acids, aspartic acid and asparagine, and it does not hit any position that has been shown to be important for zinc finger formation. Eskier et al. [34] reported that a non-synonymous substitution F234L (position according to Figure 1b, mutation not shown), identified based on analysis of ~30,000 SARS-CoV-2 genomes and located within the Nsp14 zinc finger 1, was associated with an increased genome-wide mutational load, but so were the other two identified nucleotide substitutions that were synonymous and therefore not affecting the corresponding protein sequence. 

In the relatively short and conservative Nsp10 protein of SARS-CoV-2, naturally occurring mutations are rare (Figure S1). Artificially introduced mutations in many of the evolutionarily highly conserved amino acid positions in Nsp10 have been shown to lead to reduced fidelity [65] or even be lethal to coronaviruses by interfering with viral replication [8,37]. A naturally occurring amino acid substitution R134N, detected by analysis of ~1000 genomes of SARS-CoV-2 B.1.617, is neutral and not under selective pressure [58]. 

Based on the conservative nature of components of the ExoN complex, we predict that any inhibitor for this enzymatic function should have broad-spectrum inhibitory potential for most current and possibly future strains of SARS-CoV-2.

### 3.2. Excision of Nucleotide Analogues from RNA by SARS-CoV-2 ExoN

In our previous study, we reported that Tenofovir-terminated RNA showed high resistance toward excision by the SARS-CoV-2 ExoN compared to RNA terminated with Remdesivir, Molnupiravir, Sofosbuvir or Favipiravir at the 3′ end [41]. We also demonstrated that the triphosphate forms of numerous nucleotide analogues with different structural features can be incorporated into RNA by the SARS-CoV-2 RdRp complex and terminate RNA extension with varying efficiency in either immediate or delayed fashion [46,47]. Here, with the goal of providing insights into the design of viral polymerase inhibitors that could evade the exonuclease proofreading function, we systematically investigated the chemical or structural properties of selected nucleotide analogues for exonuclease excision. 

We focus on nucleotides and nucleotide analogues with sugar modifications, such as lack of the 2**′**-OH, lack of the 3**′**-OH, or lack of both 2**′**- and 3**′**-OH, as well as base-modified nucleotides. The nucleotide analogues lacking the 2**′**-OH group include 2**′**-deoxycytosine-5**′**-triphosphate (2**′**-dCTP), 2**′**-fluoro-2**′**-deoxycytosine-5**′**-triphosphate (2**′**-F-dCTP), 2**′**-O-methylcytosine-5**′**-triphosphate (2**′**-OMe-CTP) and 2**′**-amino-2**′**-deoxyuridine-5**′**-triphosphate (2**′**-NH_2_-2**′**-dUTP). Two nucleotide analogues lacking a 3**′**-OH group, Cordycepin-5**′**-triphosphate (3**′**-dATP) and the acyclic nucleotide analogue Cidofovir diphosphate (Cid-DP), were evaluated. We also investigated nucleotide analogues lacking both 2**′**- and 3**′**-OH groups: the dideoxynucleoside-5**′**-triphosphates (ddATP, ddCTP and ddGTP), a ddUTP derivative (Stavudine-5**′**-triphosphate, Sta-TP), Zidovudine-5**′**-triphosphate (AZT-TP), as well as another acyclic nucleotide analogue, Tenofovir diphosphate (Tfv-DP). Two nucleotides with modifications at the 5-position of uridine were investigated, biotin-16-aminoallyl-2**′**-deoxyuridine-5**′**-triphosphate (Biotin-16-dUTP) and biotin-16-aminoallyl-uridine-5**′**-triphosphate (Biotin-16-UTP). Cordycepin (3**′**-deoxyadenosine) has been shown to have biological activities in a wide variety of disease processes [66] but displays some toxicity due to off-target effects on mitochondrial RNA polymerases [67]. We synthesized template-loop-primers with the different nucleotide analogues mentioned above at their 3**′** ends to perform the following exonuclease assays.

As shown in Figure 2, adenosine- (a), Cordycepin- (d), dideoxyadenosine- (g) or Tenofovir-terminated RNA (j) were separately incubated with the SARS-CoV-2 pre-assembled exonuclease complex (Nsp14/Nsp10) at 37 °C for 0 and 10 min, and the results were analyzed by MALDI-TOF mass spectrometry. The spectra in the middle (Figure 2b,e,h,k) reflected the molecular weights of the corresponding intact RNAs. After 10 min of exonuclease treatment, the RNA products were re-analyzed by MS, and the results are shown in Figure 2c,f,i,l. As an example, the peak at 8168 Da corresponds to the Cordycepin-terminated RNA before exonuclease treatment (Figure 2e). Exonuclease activity caused nucleotide cleavage from the 3′-end of the Cordycepin-terminated RNA, as shown by the eight lower molecular weight fragments corresponding to cleavage of 5–13 nucleotides (Figure 2f), with about 10% intact RNA remaining (peak at 8175 Da), indicating some level of ExoN resistance. Similarly, ddA- and Tenofovir-terminated RNAs were analyzed before (Figure 2h,k) and after ExoN treatment (Figure 2i,l). Approximately 60% and 55% of intact RNAs were observed, respectively (Figure 2i,l), indicating that both nucleotide analogues have substantial ExoN resistance. As a control, the intact adenosine-terminated RNA peak (around 8183 Da, Figure 2b) was not observed after treatment (Figure 2c), indicating that the natural nucleotide A is completely removed by the Nsp14/10 complex. By comparing spectra (Figure 2c,f,i,l), we concluded that ddA- and Tenofovir-terminated RNAs exhibit high resistance toward ExoN cleavage while Cordycepin-terminated RNA displays moderate resistance.

A previous study suggested that 3′-deoxynucleotide analogues could potentially resist exonuclease excision [11]. Moreover, 3′-deoxyadenosine-5′-triphosphate (Cordycepin TP) has been demonstrated to be incorporated efficiently and terminate RNA synthesis by the SARS-CoV-2 RdRp [66]. To confirm whether 3′-dA can evade exonuclease activity, the exonuclease resistance of Cordycepin-terminated RNA was further evaluated at different incubation times. Adenosine- and Cordycepin-terminated RNAs were treated with exonuclease for 0, 5, 10 or 15 min (Figure S2). As incubation time increases, a reduced amount of the Cordycepin-extended intact RNA peak (at ~8168 Da) is observed, while the 14 nucleotide-long RNA fragment peak (at ~4388 Da) becomes increasingly dominant (Figure S2g–j). In contrast, adenosine-terminated RNA was rapidly degraded by exonuclease (Figure S2c–e). Thus, this result indicates that 3′-dA only has some resistance to ExoN. 

Figure 3 and Figure S3 present the SARS-CoV-2 exonuclease results for uridine- and uridine analogue-terminated RNAs. The MALDI-TOF MS spectra of RNAs extended with 2′-NH_2_-2′-dUTP, Zidovudine-TP, Stavudine-TP, Biotin-16-UTP and Biotin-16-dUTP are shown in Figure 3e,h,k and Figure S3b,e. After incubation with the SARS-CoV-2 Nsp14/10 complex, only Zidovudine- and Stavudine-terminated RNAs retain ~40% and 50% of their respective intact RNA peaks (Figure 3i,l). These results indicate that Zidovudine- and Stavudine-terminated RNAs have substantial exonuclease resistance. In the control spectrum of exonuclease-treated uridine-extended RNA (Figure 3c), no intact RNA was observed, with the majority of fragments being 14–15 nucleotides long, indicated by the major peaks at 4411 Da and 4718 Da. After treatment with ExoN, the intact peak for the 2′-NH_2_-2′-dU-terminated RNA is eliminated, with mainly fragments of 18–21 nucleotides remaining (Figure 3f), indicating very low resistance to ExoN. The Biotin-16-U- and Biotin-16-dU-terminated RNAs (Figure S3c,f) also displayed very low resistance toward ExoN activity, with the intact RNA peak completely eliminated, with predominantly 18–21 nucleotide long fragments remaining.

The results for guanosine-, ddG-, Cidofovir- and ddC-terminated RNAs are shown in Figure 4. In the presence of SARS-CoV-2 ExoN, ddG-terminated RNA shows the most ExoN resistance with predominantly the intact RNA peak (8167 Da) remaining (Figure 4f). The ddC-terminated RNA displayed a substantial level of ExoN resistance but less than ddG, as indicated by the amount of intact RNA (8147 Da) remaining (Figure 4l). Cidofovir-terminated RNA had moderate resistance to ExoN excision, with even less of the intact RNA peak (8141 Da) remaining (Figure 4i). As expected, the control G-terminated RNA was completely digested by ExoN (Figure 4c).

The results for RNAs terminated with cytosine and three additional cytosine analogue-terminated synthetic RNAs were treated with SARS-CoV-2 exonuclease and are depicted in Figure S4. After treatment with ExoN for 10 min, the intact RNA peaks measured at time 0 (Figure S4e,h,k) were no longer observed for 2′-dC-, 2′-F-2′-dC- and 2′-OMe-C-terminated RNAs (Figure S4f,i,l). Compared to the control result for cytosine-terminated RNA (Figure S4c), these cytosine analogue-terminated RNAs did not demonstrate any notable resistance to exonuclease activity (Figure S4f,i,l).

**Figure 5:**
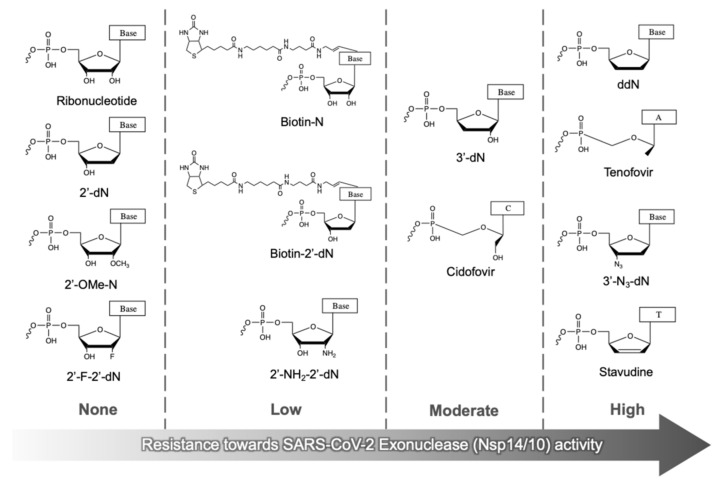
**Summary of generalized nucleotide analogue structures at the 3′ terminus of RNA organized based on their resistance towards SARS-CoV-2 exonuclease activity**. The nucleotide analogues fall into four groups showing increasing resistance to ExoN excision from left to right. N indicates any tested nucleobase.

The above results indicate that the structure of the nucleotide analogue at the 3′ terminus of RNA plays an essential role in its excision by the SARS-CoV-2 exonuclease complex. In Figure 5, the nucleotide analogues analyzed in this study are ranked in ascending order based on their resistance to exonuclease cleavage in our molecular assay. The nucleotide analogues such as 2′-dN, 2′-OMe-N and 2′-F-2′-dN, where N represents any of the nucleobases tested here, as well as the natural ribonucleotides, when present at the 3′ end of RNA, are most easily excised by ExoN. While 2′-NH_2_-2′-dN- and analogues with modifications on the base (Biotin-N and Biotin-2′-dN) provide low resistance to ExoN, 3′-deoxynucleotide analogues (3′-dN and Cidofovir) at the 3′ position of the RNA display moderate ExoN resistance. Most importantly, appreciable resistance to ExoN was observed for ddN-, 3′-N_3_-2′-dN-, Tenofovir- and Stavudine-terminated RNAs. These results also indicate that the polarity of the modification group on the sugar ring of the nucleotide analogue attached at the 3′ end of the RNA may have an effect on exonuclease cleavage. Nucleotide analogues containing the 2′- and 3′-OH (polar groups) show no resistance towards excision. However, increasing the hydrophobicity at both the 2′ and 3′ positions of the sugar ring (e.g., ddG, Stavudine and Tenofovir) results in the highest resistance to ExoN excision from RNA. 

The nucleotide analogues displaying higher resistance to exonuclease excision may help guide the further development of nucleotide analogues with particular structural features for potential coronavirus therapeutics. The ATP analogue Tenofovir-DP, the UTP analogues Stavudine-TP and AZT-TP are obligate terminators of the RdRp extension reaction though less efficiently incorporated than their corresponding natural nucleotides [46,47]. As demonstrated in this work, all three molecules resist excision by the SARS-CoV-2 ExoN to a substantial extent. The dideoxynucleotides also have significant resistance to excision by ExoN. Docking studies performed by Wu et al., indicated that ddA (Didanosine), ddC (Zalcitabine) and Stavudine had comparable binding energies for the active site of SARS-CoV-2 RdRp [68]. The structural modifications identified here will assist in the design of potentially efficient terminators of the RdRp that are also resistant to ExoN. Among the nucleotide analogues we analyzed, even those with substantial resistance to ExoN still showed some excision at longer incubation times. One approach to efficiently inhibit SARS-CoV-2 is the use of combination drug regimens involving distinct RdRp and ExoN inhibitors, as demonstrated in vitro by Wang et al. [41]. Another more challenging option is the development of a single nucleotide analogue that is relatively well incorporated by RdRp and sufficiently resistant to ExoN excision. 

## Data Availability

Not applicable.

## Supplementary Materials

The following are available online at https://www.mdpi.com/article/10.3390/v14071413/s1, Figure S1: Protein sequence conservation in the Nsp10 in representative beta *Coronaviridae* strains, Figure S2: SARS-CoV-2 exonuclease activity with adenosine- or Cordycepin-terminated RNA for different incubation times, Figure S3: SARS-CoV-2 exonuclease activity with uridine analogue-terminated RNA, Figure S4: SARS-CoV-2 exonuclease activity with cytosine- or cytosine analogue-terminated RNA.
