# Supplementary material for: Identifying Structural Features of Nucleotide Analogues to Overcome SARS-CoV-2 Exonuclease Activity
Source: Viruses. 2022 Jun 28;14(7):1413. doi: 10.3390/v14071413 (PMC9324094; doi:10.3390/v14071413)
Supplement: Supplementary file 1 [file viruses-14-01413-s001.zip › viruses-1777451- supplementary.pdf]

Identifying structural features of nucleotide analogues to overcome SARS-CoV-2 exonuclease activity

Xuanting Wang[1,2], Chuanjuan Tao[1,2], Irina Morozova[1,2], Sergey Kalachikov[1,2], Xiaoxu Li[1,2], Shiv Kumar[1,2], James J. Russo[1,2], Jingyue Ju[1,2,3,*]

1 Center for Genome Technology and Biomolecular Engineering, Columbia University, New York, NY 10027; xw2467@columbia.edu (X.W.); ct2439@columbia.edu (C.T.); im198@columbia.edu (I.M.); sk363@columbia.edu (S.Ka.); lx2109@columbia.edu (X.L.); sk3765@columbia.edu (S.Ku.); jjr4@columbia.edu (J.J.R.); dj222@columbia.edu (J.J.).

2 Department of Chemical Engineering, Columbia University, New York, NY 10027

3 Department of Molecular Pharmacology and Therapeutics, Columbia University, New York, NY 10032

* Correspondence: dj222@columbia.edu

Supplementary Material

Supplementary Figures

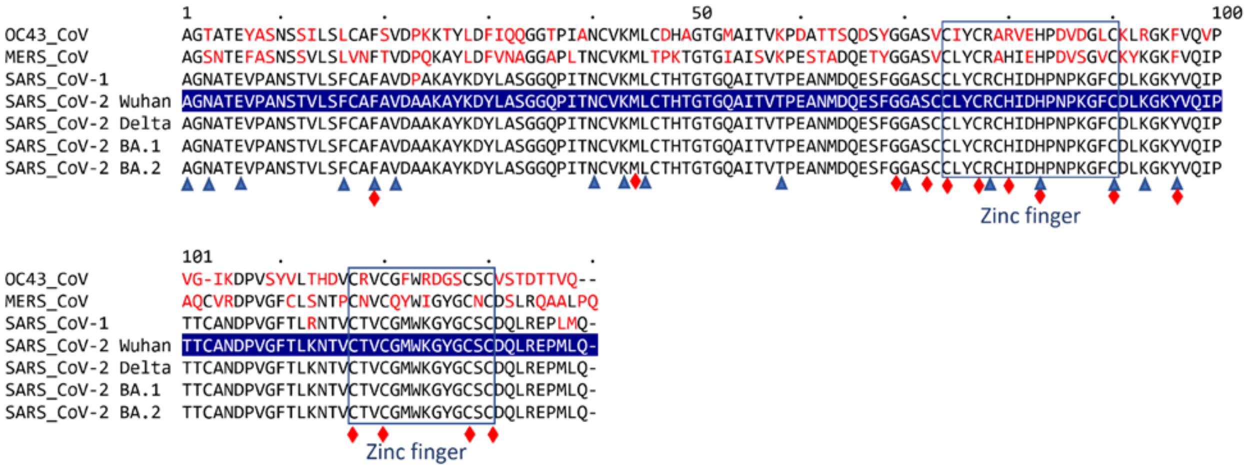

Figure S1. Protein sequence conservation in the Nsp10 in representative beta *Coronaviridae* strains. The Nsp10 of SARS-CoV-2 Wuhan-Hu-1 variant was used as a reference: amino acid differences compared to the reference sequence are highlighted in red. Zinc finger regions are boxed [1]. Residues interacting with Nsp14 are marked by blue triangles [2,3], the most important functional residues and those where mutations abolish the ExoN function or are lethal to SARS-CoV are shown as red diamonds [2-4]. The sequences were retrieved from GenBank: OC43-CoV (common cold; accession number YP_009924328), MERS-CoV (YP_009047238), SARS-CoV-1 (JX163928), and variants of SARS-CoV-2: Wuhan-Hu-1 (YP_009742617, original strain isolated in 2019), Delta (OM990852), and Omicron BA.1 (ON141240) and BA.2 (ON553707). Protein alignment was built using Clustal Omega [5,6], visualized by MView [6,7], and annotated.

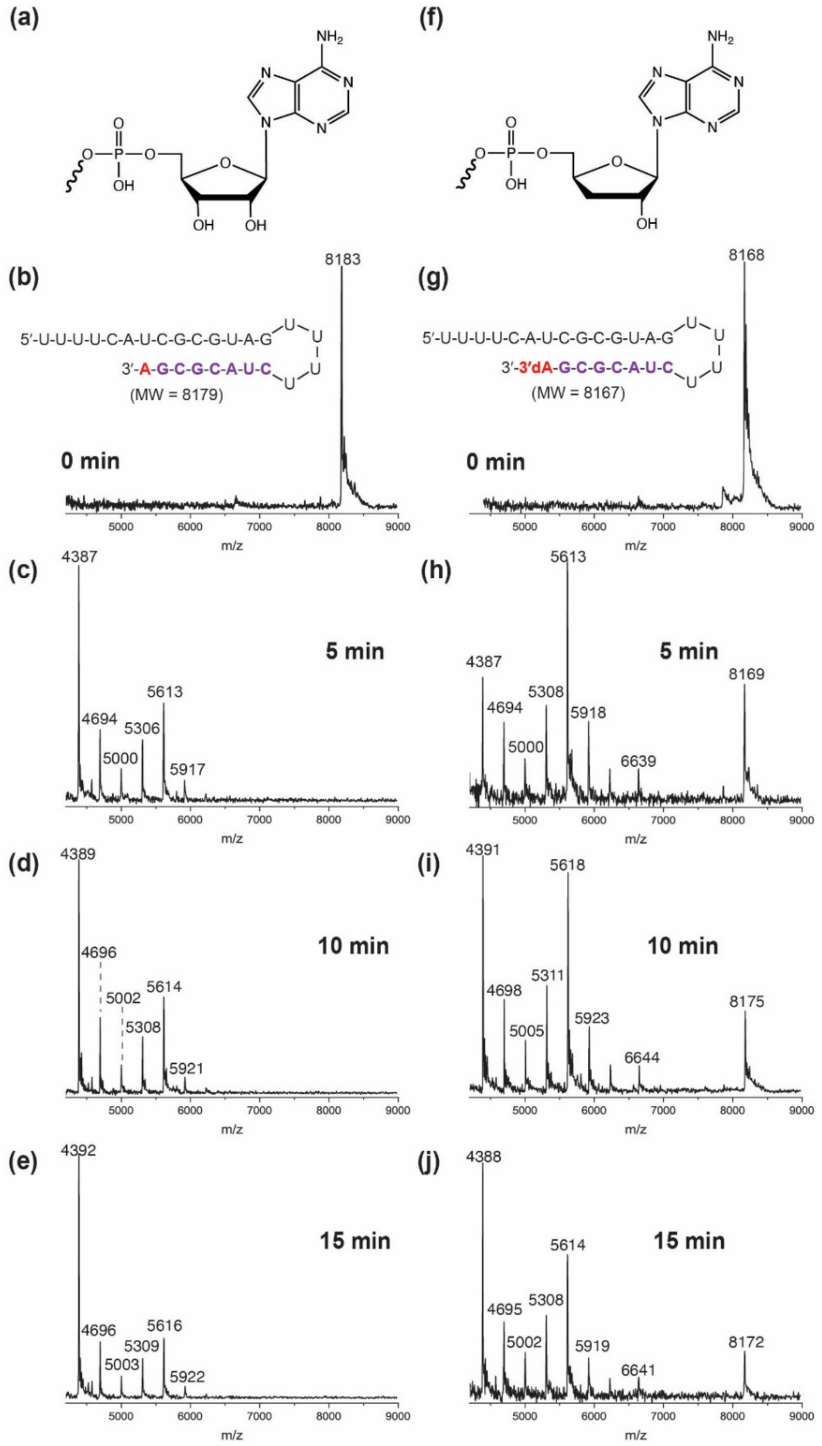

Figure S2. SARS-CoV-2 exonuclease activity with adenosine- or Cordycepin-terminated RNA for different incubation times. A mixture of 500 nM of adenosine-terminated template-loop-primer (sequences shown at the top of **b**) and SARS-CoV-2 pre-assembled exonuclease complex (Nsp14/Nsp10) was incubated at 37 °C for 5 min (**c**), 10 min (**d**), or 15 min (**e**). A mixture of 500 nM of Cordycepin-terminated template-loop-primer (sequences shown at the top of **g**) and SARS-CoV-2 pre-assembled exonuclease complex (Nsp14/Nsp10) was incubated at 37 °C for 5 min (**h**), 10 min (**i**), or 15 min (**j**). The intact RNAs (**b, g**) and their respective exonuclease reaction products (**c-e, h-j**) were analyzed by MALDI-TOF MS. The signal intensity was normalized to the highest peak.

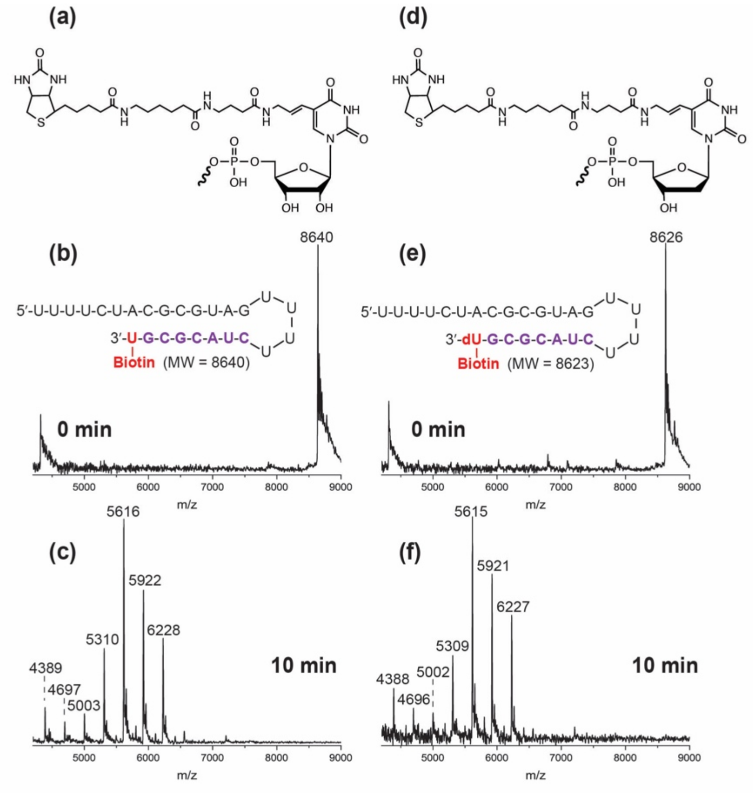

Figure S3. SARS-CoV-2 exonuclease activity with uridine analogue-terminated RNA. A mixture of 500 nM template-loop-primer terminated at its 3′ end with either Biotin-16-U (**a**) or Biotin-16-dU (**d**) (sequences shown in **b, e**) and SARS-CoV-2 pre-assembled exonuclease complex (Nsp14/Nsp10) was incubated at 37 °C for 10 minutes. These intact RNAs (**b, e**) and their respective exonuclease reaction products (**c, f**) were analyzed by MALDI-TOF MS. The signal intensity was normalized to the highest peak.

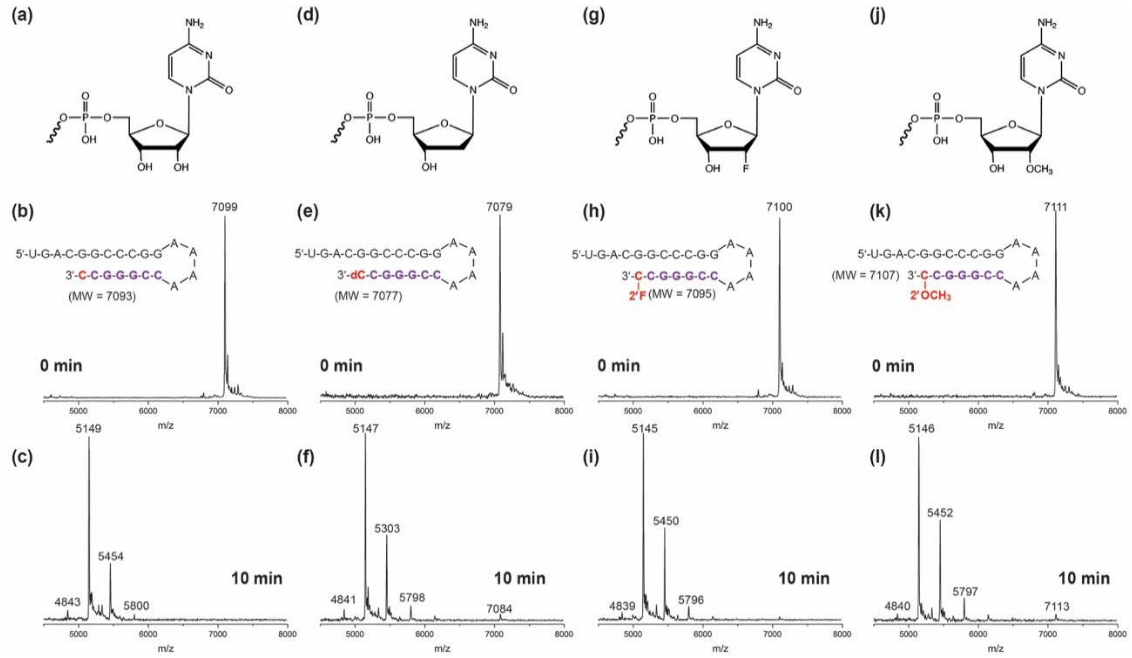

Figure S4. SARS-CoV-2 exonuclease activity with cytosine- or cytosine analogue-terminated RNA. A mixture of 500 nM of template-loop-primer terminated at its 3'end with either C (**a**), 2'-dC (**d**), 2'-F-2'-dC (**g**) or 2'-OMe-C (**j**) (sequences shown in **b, e, h, k**) and SARS-CoV-2 pre-assembled exonuclease complex (Nsp14/Nsp10) was incubated at 37 °C for 10 minutes. These intact RNAs (**b, e, h, k**) and their respective exonuclease reaction products (**c, f, i, l**) were analyzed by MALDI-TOF MS. The signal intensity was normalized to the highest peak.

Supplementary References

1. Lin, S.; Chen, H.; Chen, Z.; Yang, F.; Ye, F.; Zheng, Y.; Yang, J.; Lin, X.; Sun, H.; Wang, L.; et al. Crystal structure of SARS-CoV-2 nsp10 bound to nsp14-ExoN domain reveals an exoribonuclease with both structural and functional integrity. *Nucleic Acids Res*. **2021**, *49*, 5382–5392. https://doi.org/10.1093/nar/gkab320

2. Ma, Y.; Wu, L.; Shaw, N.; Gao, Y.; Wang, J.; Sun, Y.; Lou, Z.; Yan, L.; Zhang, R.; Rao, Z. Structural basis and functional analysis of the SARS coronavirus nsp14–nsp10 complex. *Proc. Natl. Acad. Sci. USA* **2015**, *112*, 9436–9441. https://doi.org/0.1073/pnas.1508686112

3. Bouvet, M.; Lugari, A.; Posthuma, C.C.; Zevenhoven, J.C.; Bernard, S.; Betzi, S.; Imbert, I.; Canard, B.; Guillemot, J.-C.; Lécine, P.; et al. Coronavirus nsp10, a critical co-factor for activation of multiple replicative enzymes. *J. Biol. Chem.* **2014**, *289*, 25783–25796. https://doi.org/10.1074/jbc.M114.577353

4. Moeller, N.H.; Shi, K.; Demir, Ö.; Belica, C.; Banerjee, S.; Yin, L.; Durfee, C.; Amaro, R.E.; Aihara, H. Structure and dynamics of SARS-CoV-2 proofreading exoribonuclease ExoN. *Proc. Natl. Acad. Sci. USA* **2022**, *119*, e2106379119. https://doi.org/10.1101/2021.04.02.438274

5. Sievers, F.; Wilm, A.; Dineen, D.; Gibson, T.J.; Karplus, K.; Li, W.; Lopez, R.; McWilliam, H.; Remmert, M.; Söding, J.; et al. Fast, scalable generation of high-quality protein multiple sequence alignments using Clustal Omega. *Mol. Syst. Biol.* **2011**, *7*, 539. https://doi.org/10.1038/msb.2011.75

6. Madeira, F.; Pearce, M.; Tivey, A.R.N.; Basutkar, P.; Lee, J.; Edbali, O.; Madhusoodanan, N.; Kolesnikov, A.; Lopez, R. Search and sequence analysis tools services from EMBL-EBI in 2022. *Nucleic Acids Res.* **2022**. online ahead of print. https://doi.org/10.1093/nar/gkac240

7. Brown, N.P.; Leroy, C.; Sander, C. MView: A web-compatible database search or multiple alignment viewer. Bioinformatics 1998, 14, 380–381. https://doi.org/10.1093/bioinformatics/14.4.380
